# Regulation of Antibiotic Resistance Genes on Agricultural Land Is Dependent on Both Choice of Organic Amendment and Prevalence of Predatory Bacteria

**DOI:** 10.3390/antibiotics13080750

**Published:** 2024-08-10

**Authors:** Anna Karin Rosberg, Maria João Silva, Cecilie Skøtt Feidenhans’l, Eddie Cytryn, Edouard Jurkevitch, Rolf Lood

**Affiliations:** 1 Microbial Horticulture Laboratory, Department of Biosystems and Technology, Swedish University of Agricultural Sciences, 234 56 Alnarp, Sweden; anna.karin.rosberg@slu.se; 2 Department of Clinical Sciences Lund, Division of Infection Medicine, Lund University, 221 84 Lund, Sweden; maria_joao.silva@med.lu.se; 3 Department of Biomedicine, Faculty of Health, Aarhus University, 8000 Aarhus, Denmark; cecilief@biomed.au.dk; 4 Institute of Soil, Water and Environmental Sciences, Volcani Center, Agricultural Research Organization, P.O. Box 15159, Rishon LeZion 7528809, Israel; eddie@volcani.agri.gov.il; 5 Department of Plant Pathology and Microbiology, Faculty of Agriculture, Food and Environment, The Hebrew University of Jerusalem, Rehovot 7610001, Israel; edouard.jurkevitch@mail.huji.ac.il

**Keywords:** soil, manure, sewage sludge, antibiotic resistance, BALO, tetracycline, vancomycin

## Abstract

Antibiotic resistance genes (ARGs) are widespread in the environment, and soils, specifically, are hotspots for microorganisms with inherent antibiotic resistance. Manure and sludge used as fertilizers in agricultural production have been shown to contain vast amounts of ARGs, and due to continued applications, ARGs accumulate in agricultural soils. Some soils, however, harbor a resilience capacity that could depend on specific soil properties, as well as the presence of predatory bacteria that are able to hydrolyse living bacteria, including bacteria of clinical importance. The objectives of this study were to (i) investigate if the antibiotic resistance profile of the soil microbiota could be differently affected by the addition of cow manure, chicken manure, and sludge, and (ii) investigate if the amendments had an effect on the presence of predatory bacteria. The three organic amendments were mixed separately with a field soil, divided into pots, and incubated in a greenhouse for 28 days. Droplet digital PCR (ddPCR) was used to quantify three ARGs, two predatory bacteria, and total number of bacteria. In this study, we demonstrated that the choice of organic amendment significantly affected the antibiotic resistance profile of soil, and promoted the growth of predatory bacteria, while the total number of bacteria was unaffected.

## 1. Introduction

Soil harbors a vast amount of microorganisms and is a large reservoir of antibiotic resistance genes (ARGs). Globally, hotspots for ARGs in soils are commonly located in densely populated areas, such as the eastern United States, Western Europe, South Asia, and East China. There is a higher abundance in agricultural habitats [1], although in general, the accumulation is largely affected by exposure to manure amendment, sewage, treated wastewater, and industrial contamination. While the presence of ARGs in soils has increased since the application of antibiotics used against human and animal diseases [2], antibiotic resistance has always occurred as a response, providing a competitive advantage to naturally occurring antibiotics [3]. However, extensive use of antibiotics, both within healthcare and agriculture, combined with low uptake in humans and animals, can potentially result in increased concentrations in the soil in regions that apply manure and sewage sludge in agricultural production [2]. Organic cow manure has earlier been described to contain significant levels of ARGs against kanamycin, chloramphenicol, beta-lactams, and tetracyclines [4]. In general, there is a strong correlation between the high usage of antibiotics in cattle and the abundance of ARGs in their manure. Importantly, the abundance of ARGs can be over 28,000 times higher in manured soil compared to un-manured soil [5]. Manure has, therefore, been described as a potential hotspot for the spread of antibiotic resistance [6]. Long-term experiments with annual applications of cow manure showed a linear increase in ARGs, with a clear connection between ARG abundance and manure application rate [7]. Manure coming from different farm animals has been shown to contain significantly different diversities and abundances of ARGs. This is partly due to differences in the types of antibiotics provided as well as animal-specific microbiome composition, resulting in different scopes of ARGs, with chicken and pig manure harboring higher levels of ARGs compared to cow manure [8]. The abundance of ARGs in manure is, however, dependent on where the animals are bred, with large variations both between and within countries [5]. The use of sewage sludge originating from wastewater treatment plants (WWTP) in agricultural production is another means of meeting crop nutrient demands that has also been associated with increased abundances of ARGs in soils [9,10,11,12]. In 2010, it was estimated that 39% of the sewage sludge produced in the European Union member states was recycled in agriculture as a source of nutrients and organic matter [13]. While the use of sludge is encouraged [14], it comes with many restrictions, primarily targeting human and environmental health in terms of the potential accumulation of heavy metals and organic contaminants. However, antimicrobial resistance is a growing concern, and in the latest suggestions for improvements to the EU directive concerning urban wastewater, all member states shall sample and monitor WWTP with <100,000 population equivalents for antimicrobial resistance [15].

Antibiotic resistant bacteria are numerous, and the most critical for human health are considered to be part of the ESKAPE bacteria (*Enterococcus faecium*, *Staphylococcus aureus*, *Klebsiella pneumoniae*, *Acinetobacter baumannii*, *Pseudomonas aeruginosa*, and *Enterobacter* species) [16], with carbapenem and extended spectrum beta-lactamase (ESBL) resistance being some of the more significant antibiotic resistances currently spreading. These resistance genes, however, currently exist at low relative abundances in environmental samples, although some WWTPs have been described as sources of ESBL-spread [17]. Intestinal bacteria are known carriers of mobile genetic elements that are vectors for genetic exchange. Thus, increased fertilization with manure and sewage sludge containing both intestinal bacteria and antibiotics, combined with the already large soil reservoir of ARGs, poses increasing risks of ARGs spreading to pathogens of clinical importance through lateral and horizontal gene transfer [18]. Trials investigating the presence of ARGs in crops produced in fields amended with manure have shown an overlap of genes between the edible parts of the crops and the manure, indicating an interconnectedness between the resistomes of plants and the production environment [12,19]. This suggests a potential route of ARG transfer from the environment into the human microbiome, with potential implications on human health.

While there is plenty of research supporting the accumulation of ARGs in soil as a consequence of manure and sewage sludge use, the results can be contradictory. Soil properties, such as texture and pH, are recognized factors impacting the accumulation of ARGs [20]. Some antibiotics have a high adsorption capacity to soil particles, reducing their rates of degradation, and they therefore tend to accumulate in soils to a higher degree [21]. Adsorption capacity depends on soil quality characteristics, such as organic matter content, cation exchange capacity, clay surface charge, and pH. Tetracycline, for example, has been shown to adsorb more on acidic soils with a high clay content [21]. The already present soil microbial communities have also been suggested to affect the fate of the added ARGs, or antimicrobial resistance (AMR), by outcompeting added bacteria harboring resistance genes [7,22]. Soil properties, antibiotic properties, and bacterial community characteristics are thus factors that could affect the persistence of ARG in soil. Predatory bacteria, including *Bdellovibrio* and like organisms (BALOs), comprise a group of Gram-negative bacteria that prey on other bacteria, either through attachment to the prey bacteria, local lysis of the cell wall, and extraction of the internal macromolecules, or through prey invasion and lysis from the inside. Their function in soil has been described as that of trophic controllers [23], both through the hydrolysis of live bacteria and through their significant secretion of a plethora of hydrolases [24], including proteases [25,26] and nucleases [27]. Two of the more studied predatory bacterial families are *Bdellovibrionaceae* (genus: *Bdellovibrio*) and *Bacteriovoracaceae* (genus: *Bacteriovorax* and *Peridibacter*). Both are significantly smaller than most bacteria, facilitating an intracellular lifestyle as well as being mobile through the presence of a flagellum. Both *Bdellovibrio* and *Bacteriovorax* have been demonstrated to lyse several important Gram-negative pathogens, even antibiotic-resistant bacteria, including *Pseudomonas aeruginosa*, *Serratia marcescens*, *Acinetobacter baumannii*, *E. coli*, and *Salmonella* [28,29,30,31,32]. Specifically, several BALOs have been isolated from soil and have been demonstrated to hydrolyze soil microbes [33]. While their ability to kill specific pathogens have been demonstrated, their ability to shape the microbiota in soil, or, more specifically the soil resistome, has, to our knowledge, not been investigated.

The objective of this study was to investigate if the antibiotic resistance profile of the soil microbiota (16S rRNA) could be differently affected by the addition of cow manure, chicken manure, and sludge. Due to the low abundance of ESBL resistance genes in environmental samples, they are poor candidates as biomarkers for the generalized spread of antibiotic resistance. Therefore, to investigate the flux of resistance genes constituting soil resistance (i.e., the genes are not able to ‘colonize’ the soil) and soil resilience (i.e., the genes are able to establish but are rapidly removed from the system), we monitored a set of chosen antibiotic resistance genes previously shown to be common in soil and water [34,35,36,37]. These can be found even in soils with limited impact from humans, which means that they are indigenous to the soil [38]. The purpose of these genes would be to use them as general biomarkers for resistance gene fluctuations in the soil rather than focusing on their clinical impact. Due to the prevalence and biological function of predatory bacteria, we hypothesize that they constitute part of the ability of soil to buffer the spread of ARGs originating from exogenously applied sources, such as manure, both through the resistance and resilience of the soil. Furthermore, we hypothesize that different types of organic amendments may differently affect the microbiota, predatory bacteria and antibiotic resistance profiles of the soil. Herein, we have shown that the choice of organic amendments significantly impacts the antibiotic resistance profile of soil and promotes the growth of predatory bacteria. Importantly, the prevalence of predatory bacteria correlates with ARGs, demonstrating an interaction between the two.

## 2. Results

### 2.1. Choice of Manure Drives Prevalence of Unique Antibiotic Resistance Patterns in the Soil Microbiota

**Figure 1:**
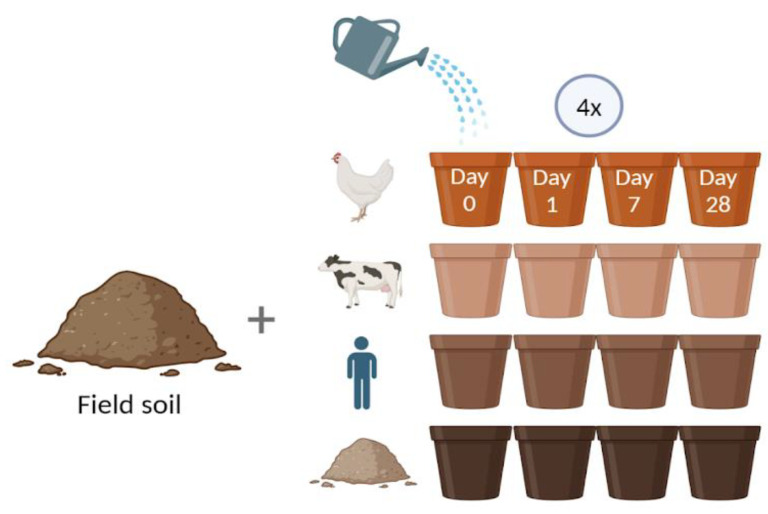
Schematic overview of the study design. Soil from organic production was added to the pots and complemented with different types of organic amendments: chicken manure, cow manure, and sludge. All pots were watered, covered with black plastic, and incubated in a greenhouse for 28 days. Samples were taken at four time points with four biological replicates. This image was created with BioRender.com accessed on 4 July 2024.

**Figure 2:**
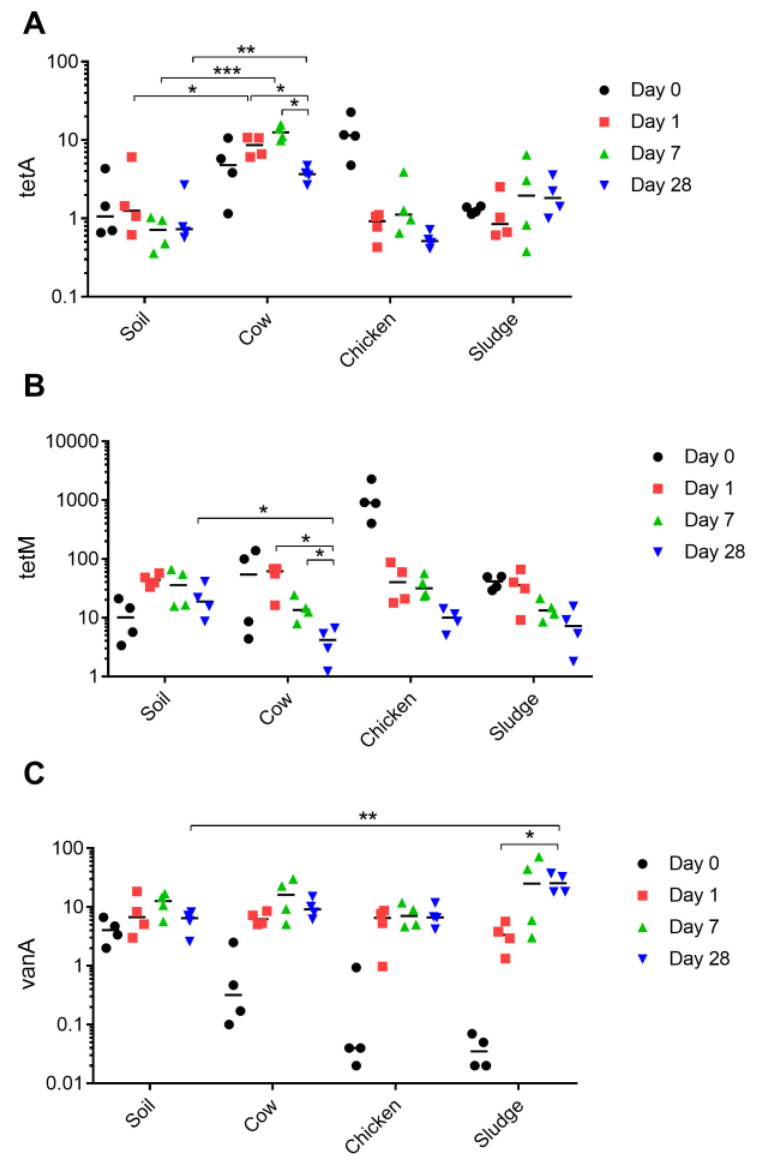
Prevalence of antibiotic resistance genes in soil exposed to different organic manure. DNA was extracted from soil exposed to different manure (cow, chicken, or sludge) for a time span of 0, 1, 7, or 28 days. Gene copies of (**A**) *tetA*, (**B**) *tetM*, and (**C**) *vanA* were determined through ddPCR and reported as copies per gram soil. Samples from day 0 represent the additive only (e.g., manure). Each mark (i.e., black dot, red square, and green or blue triangle) represents a true biological sample for which three technical replicates were conducted. For each sample, four biological replicates were taken (i.e., four marks per time point), and is displayed as the four different time points for each amendment on the x-axis, and number of gene copies on the y-axis. * *p* < 0.05, ** *p* < 0.01, *** *p* < 0.001.

To investigate if the abundance of the targeted ARGs in the soil microbiota could be affected by the addition of organic amendments, cow manure, chicken manure, and sludge were added to the soil from an organic farm and incubated for up to 28 days (Figure 1). The addition of cow manure significantly increased the prevalence of *tetA* following amendment, and this increase persisted till the end of the experiment on day 28 (Figure 2A). In contrast, the presence of *tetM* was significantly reduced in soil treated with cow manure as compared to the soil control (Figure 2B). In the sludge-amended soil, a significant increase in the abundance of *vanA* was only observed at the endpoint of the experiment (Figure 2C). The addition of chicken manure did not significantly affect the prevalence of any of the resistance genes investigated. Measurements at day 0 represent the organic amendment itself (except in the soil control). All organic amendments contained lower quantities of resistance genes per gram than the soil itself, with the exception of chicken manure, which contained high levels of *tetA* and *tetM*.

**Figure 3:**
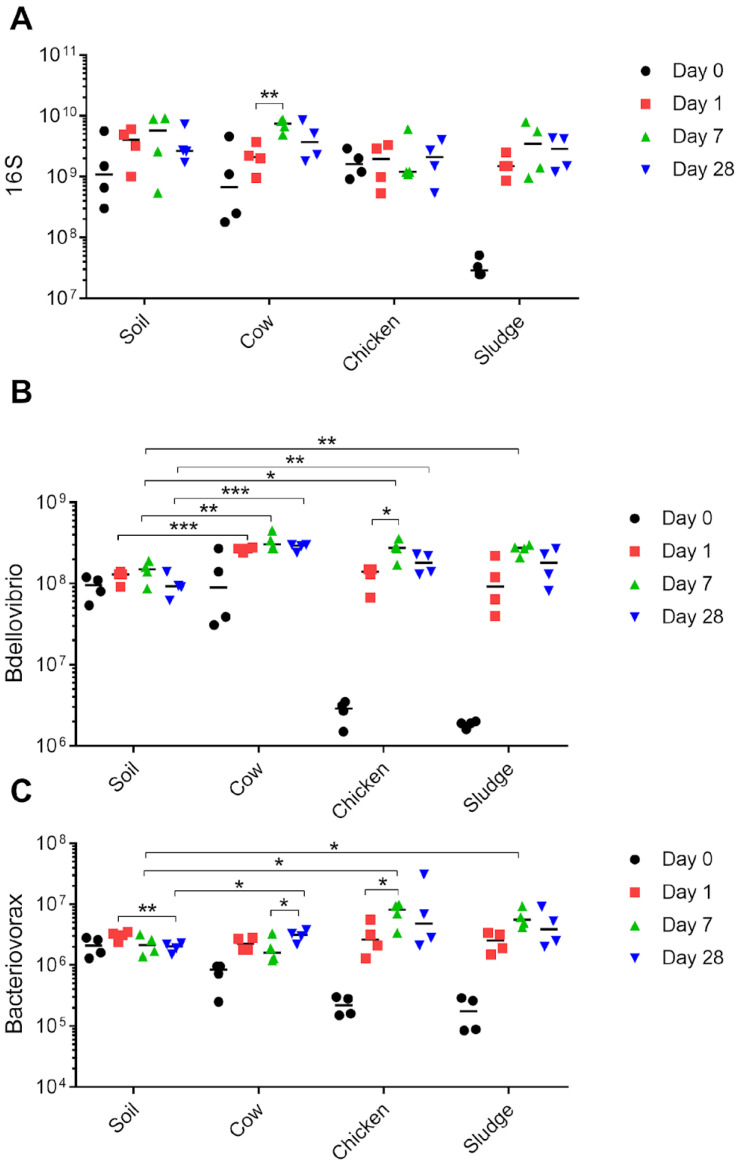
Prevalence of predatory bacteria in soil exposed to different organic manure. DNA was extracted from soil exposed to different manure (cow, chicken, or sludge) for a time span of 0, 1, 7, or 28 days. Gene copies of (**A**) *16S* rRNA of the total bacterial population, (**B**) 16S rRNA gene copies of *Bdellovibrio*, and (**C**) 16S rRNA gene copies of *Bacteriovorax* were determined through ddPCR and reported as copies per gram soil. Samples from day 0 represent the additive only (e.g., manure). Each mark (i.e., black dot, red square, green or blue triangle) represents a true biological sample for which three technical replicates were conducted. For each sample, four biological replicates were taken (i.e., four marks per time point), and are displayed as the four different time points for each amendment on the x-axis, and number of gene copies on the y-axis. * *p* < 0.05, ** *p* < 0.01, *** *p* < 0.001.

To further investigate the impact of organic amendments on the prevalence of resistance genes in soil, we analyzed the prevalence of the resistance gene in correlation to the abundance of bacteria (e.g., 16S rRNA). Minor differences could be identified for both *tetA* and *tetM*, with *tetA* significantly higher in the cow manure amended soil at day 7, compared to sludge amended soil (Figure 3A). For *tetM*, there was a reduction in prevalence between days 7–28 (chicken manure) and days 1–7 (sludge) (Figure 3B). *vanA* was the only resistance gene demonstrating significantly higher levels as compared to the soil itself at the end of the study (sludge; Figure 3C). Within the sludge-amended soil, there was also a significant increase in *vanA* prevalence between day 1 and day 28 (Figure 3C).

### 2.2. Predatory Bacteria Benefit from the Addition of Organic Amendments

**Figure 4:**
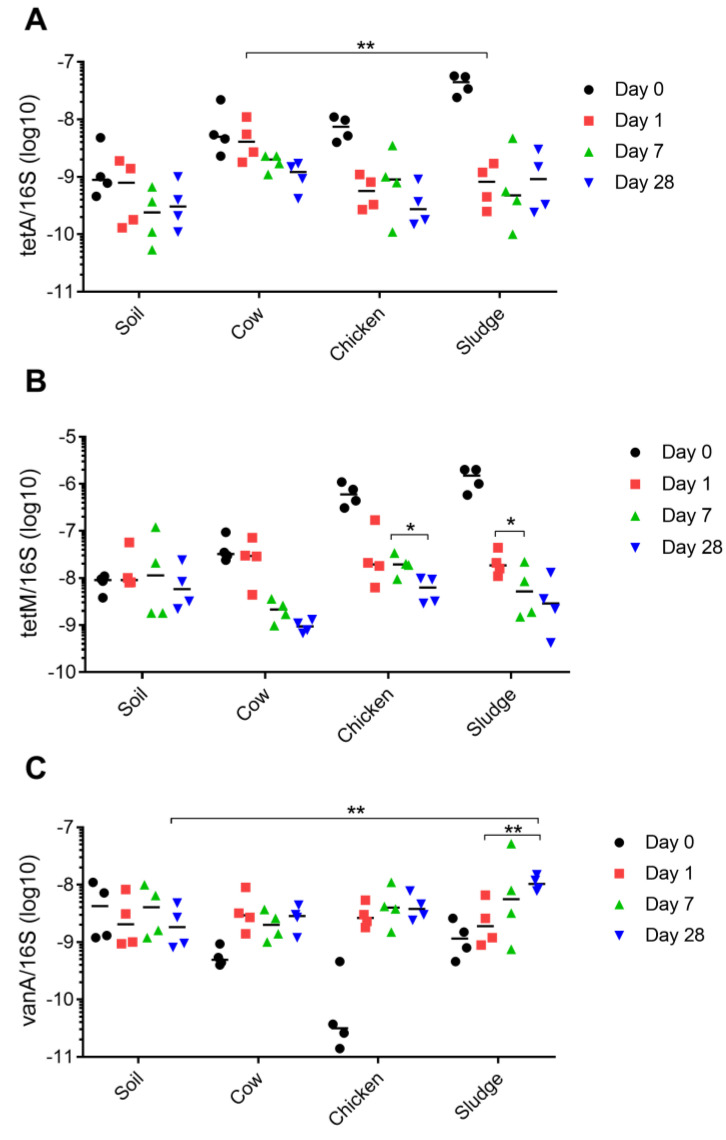
Prevalence of antibiotic resistance genes in relation to 16S. Gene copy number per gram soil (log10) of antibiotic resistance genes and 16S from all soil samples were collected at 0, 1, 7, and 28 days, and relative values are shown for (**A**) *tetA*, (**B**) *tetA*, and (**C**) *vanA*. Each mark (i.e., black dot, red square, and green or blue triangle) represent a true biological sample for which three technical replicates were conducted. For each sample, four biological replicates were taken (i.e., four marks per time point), and are displayed as the four different time points for each amendment on the x-axis, and number of gene copies on the y-axis. * *p* < 0.05, ** *p* < 0.01.

Learning that organic amendments differently impact the abundance of the three targeted ARGs, we turned to investigate the impact on the quantity of the microbiota. None of the additives significantly impacted the size of the general microbial population (Figure 4A), indicating that changes in resistance profiles are not due to an overall change in the population size of the microbiota. However, both *Bdellovibrio* and *Bacteriovorax* significantly increased their population sizes over time in all treatments (Figure 4B,C). It should be noted that *Bdellovibrio* are commonly 1–2 orders of magnitude more abundant than *Bacteriovorax*. Measurements on day 0 represent the organic amendment itself (except in the soil control). All organic amendments contained lower quantities of bacteria per gram than the soil itself.

### 2.3. Presence of Predatory Bacteria Significantly Correlate with Prevalence of ARGs

**Figure 5:**
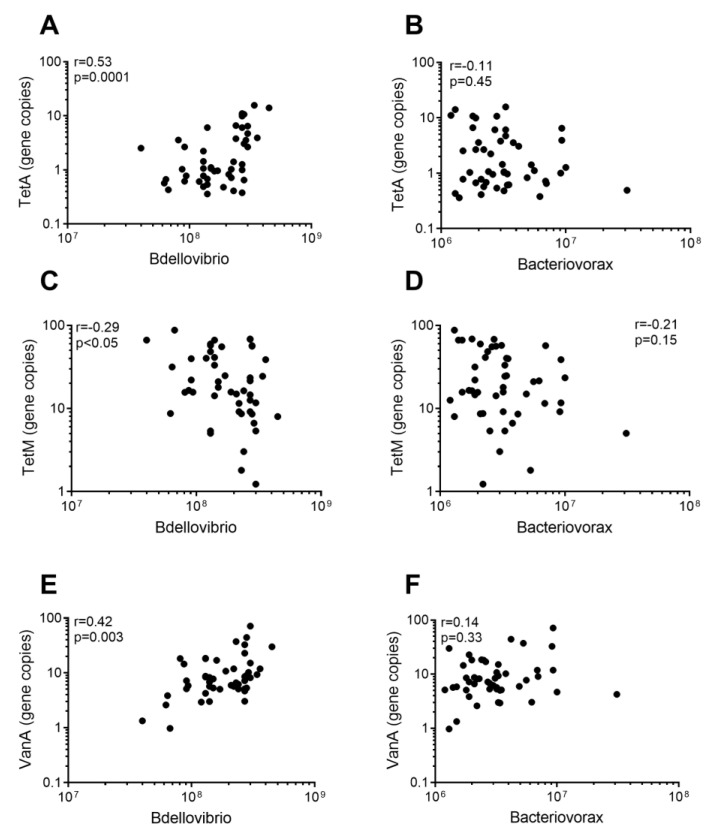
Correlations between the prevalence of antibiotic resistance genes and the 16S rDNA gene of predatory bacteria. Absolute quantities (gene copy number per gram soil) of predatory bacteria and antibiotic resistance genes from all soil samples were analyzed for correlative values between (**A**) *tetA* and *Bdellovibrio*, (**B**) *tetA* and *Bacteriovorax*, (**C**) *tetM* and *Bdellovibrio*, (**D**) *tetM* and *Bacteriovorax*, (**E**) *vanA* and *Bdellovibrio*, and (**F**) *vanA* and *Bacteriovorax*.

Hypothesizing that predatory bacteria may be able to contribute to buffer soil against invasive bacterial species, including those carrying ARGs, we investigated if absolute quantities of predatory bacteria correlated with the prevalence of ARGs. *Bdellovibrio* was positively correlated with absolute quantities of *tetA* and *vanA*, while being negatively correlated with *tetM* (Figure 5A,C,E). For *Bacteriovorax*, correlations were not significant (Figure 5B,D,F). Total bacterial abundance (16S rRNA) correlated positively (r = 0.42) with the presence of *vanA* (*p* = 0.003)(Table S1).

### 2.4. Spurway Analysis

The Spurway analysis showed differences in pH as well as several larger differences in nutrient levels between treatments (Table 1). The pH ranged from 5.45 in soil amended with sludge to 6.35 in soil amended with cow manure. Large variations were observed in, but not restricted to, nitrogen, potassium, and phosphorus levels. Soil amended with cow manure had high levels of both potassium and phosphorus (420 mg/L and 110 mg/L, respectively), while soil amended with sludge had the highest nitrogen level (NO_3_^−^: 210 mg/L).

## 3. Discussion

This study investigated the fluctuations of three clinically important ARGs in soils fertilized with three types of organic amendments and their correlations with the abundance of soil bacteria and with the predatory bacterial genera *Bdellovibrio* and *Bacteriovorax*. The resistance genes *tetM* and *tetA* were present in both cow and chicken manure, as well as in sludge, while *vanA* was not. However, the different organic amendments showed differential effects on the persistence of all three resistance genes in the incubated soil samples, which was not correlated to their initial levels in the organic amendments. Despite the rather high levels of ARGs in the initial organic amendments’ samples, the soil did not maintain these levels. While a natural dilution of the ARGs was made when the organic amendments were mixed with the soil, in most cases there was an additional decline from day 1 of incubation until day 28, indicating the resilience capacity of the soil. It should be noted that two amendments led to higher quantities of ARGs in the soil: cow manure (*tetA*) and sludge (*vanA*). The mere presence of resistance genes does, however, not easily translate into beneficial or detrimental effects, since it is highly dependent on what species are carrying the resistance genes. Several soil microbiota species carry ARGs as a means to survive microbially produced antibiotics, and their presence is thus key to retaining a functional microbiota. Previous research shows contradictory results concerning the capacity of amended ARGs to persist in the soil. In a study carried out in Spain, agricultural soils amended with sewage sludge showed an increased risk of antibiotic resistance dissemination [11], while a Swedish study investigating ARGs in soils amended with sewage sludge for nearly 40 years did not indicate any changes in the scope and intensity of the soil bacterial resistome connected to sludge applications [39]. Similarly, Radu et al. [40] showed initial increasing levels of clinically relevant ARGs after the addition of pig manure to the soil. However, at the end of the cropping season, the ARG levels returned to their initial background levels, indicating the resilience capacity of the soil. The soil quality [21], the initial abundance of ARGs in the organic amendments [5], and the inherent soil microbiome [7,22] could possibly explain such variability in accumulation potential.

In this study, rather low levels of ARGs were detected (10^0^–10^2^ gene copies per gram of soil) of the selected resistance genes. This is in contrast to some other studies demonstrating almost 2 log higher levels of tetracycline resistance genes within conventional dairy farms in the Czech Republic [41]. However, a similar study in the USA failed to even identify any *tetA* or *tetM* from soil in Nebraska [42], indicating that the site, and how the soil has been treated (e.g., crops, animals, manure, and antibiotics usage culture in the country) all affect the prevalence of resistance genes. Similarly, on a global level, Sweden is using very low quantities of antibiotics, both within health care [43] and agriculture [44], which could be an explanation for the low detection rate in our study.

Soil nutrient levels have been associated with the presence of ARGs, with positive correlations to the levels of nitrogen, phosphorus, and potassium, and organic matter content [45]. This could not be confirmed in our data, where chicken manure harbored the highest abundances of *tetM* and *tetA* genes, while the highest levels of nitrogen, phosphorus, and potassium were measured in sludge and cow manure. In regard to the elevated levels of *tetA* and *tetM* in chicken manure, it should be noted that the soil levels of these resistance genes remained low even after chicken manure amendment, demonstrating the resilience capacity of the soil to remove ARGs and return to baseline levels due to the microbial ecosystem functioning of the soil.

The addition of nutrients has previously been described to disproportionately favor the growth of predatory bacteria over non-predatory bacteria, where *Bdellovibrionales* were growing 63% faster than non-predators [23]. The authors conclude that predatory bacteria play a significant role in controlling lower trophic levels, and as such, significantly increase with added nutritional value in their niche. In support of this, we could demonstrate that the addition of organic amendments to soil is mainly affecting the predatory population compared to the non-predatory population.

Our data show that the levels of *Bdellovibrio* and ARGs correlate. However, the correlation is both positive and negative depending on the ARG (i.e., *tetA*, *tetM*, or *vanA*), indicating a complex interaction. It should be noted that even though the correlations are significant, the r values are rather low (0.11–0.53), possibly due to the complex nature of such correlations. This interaction is further complicated by the fact that the resistance genes can be removed either through predation of the prey bacteria, or through direct hydrolysis of extracellularly present ARGs through secreted nucleases. Furthermore, the microbial soil community will also be affected by the reduction of prey cells due to the expansion of predatory cells, increasing available nutrients for other non-prey bacterial species to use, and expanding their population as well. *tetA*, commonly found in *E. coli* and other Gram-negative bacteria, should constitute prey for *Bdellovibrio* and, as such, a negative correlation would be expected, i.e., the higher the quantities of *Bdellovibrio* the lower the quantities of *E. coli tetA*. We do, however, detect a positive correlation, indicating an overall increase in the prey resistance gene with increasing numbers of predatory bacteria. The complexity of prey–predator interactions in simpler systems has been evaluated, suggesting re-growth of the prey after initial reduction [46]. However, community-level analyses have revealed that the interactions are complex, with predatory bacteria affecting the general population in a density-dependent manner [47]. The molecular mechanisms underlying these predator–prey dynamics have not been fully understood, but a plastic resistance mechanism nor have suboptimal conditions for the predator’s ability to infect been suggested [48]. Further, the presence of other microorganisms (i.e., mixed microbial populations) has been demonstrated to affect the interaction of *Bdellovibrio* and its prey [49], while others have seen a less significant impact of such polymicrobial cultures [50]. In addition, others have suggested that bacterial predators (protists) may even lead to an increased prevalence of ARG and ARM due to a selective pressure for the production of antibiotics by bacteria to fight protists, and thus selection for antibiotic resistance as well [51]. Further investigation into these correlations is thus of value.

For *tetM*, we demonstrate a negative correlation to *Bdellovibrio*. However, *tetM* is mainly carried by Gram-positive bacteria that are not necessarily prey for *Bdellovibrio*. Thus, it is unlikely that the negative correlation is due to the predation of the resistant bacteria and/or the resistance genes. Rather, it may be an inverse correlation due to high levels of *tetM*-carrying Gram-positive bacteria negatively impacting the abundance of *Bdellovibrio* prey through nutritional competition. *vanA* is also mainly associated with Gram-positive bacteria (*Enterococcus* and *Staphylococcus*) and is positively correlated with the prevalence of *Bdellovibrio*. Similarly to *tetM*, this correlation is likely mediated by nutritional competition, with high levels of *Bdellovibrio* reducing several Gram-negative species, creating an opportunity for *vanA*-carrying Gram-positive bacteria to expand their population. It should, however, be noted that factors other than predatory bacteria may impact ARG abundance in the soil, among others including phages, protozoa, and competing bacteria producing toxins.

It should be noted that a limitation of our study is the singular location that soil was collected from. A different microbial composition, or resistome profile, may have impacted the specific outcomes of this study. Likewise, different concentrations of the organic amendments, lengths of incubation in soil, as well as the frequency of sampling occasions, may impact the study outcome differently. Therefore, we are only suggesting that BALOs may impact the resistome and use the specified resistance genes as biomarkers for this phenomenon. Furthermore, since we cannot in this study determine what bacteria (species) are carrying the resistance genes, we cannot ascertain the mechanisms behind the prey–predator dynamics. Mapping of the microbial community population (e.g., 16S rRNA) could shed some light on community modifications but would unfortunately not demonstrate resistance profile changes. Further studies detailing this would be of importance to better understand the flow of ARGs within soil.

In conclusion, we have demonstrated that the type of organic amendment can significantly impact the ARG profile of soil, both as an immediate effect and as a long-term effect. Further, we propose that *Bdellovibrio*, as a part of the predatory bacterial community, is part of shaping the antibiotic resistome in soil, through complex interactions. The latter, however, does need to be investigated specifically to demonstrate if the correlation identified here can be translated into causation. The mechanistic insights into how such interactions are mediated and how they would impact the spread of antibiotic resistance to human pathogens merits further investigation.

## 4. Materials and Methods

### 4.1. Soil Incubation with Organic Amendments

A sandy loam soil from an organically managed research field (Alnarp, Sweden) was collected, carefully homogenized, and dispensed in pots. For the different treatments, field soil was mixed separately with three types of organic amendments: commercially available cow and hen manures (Granngården, Malmö, Sweden), and sludge from a wastewater treatment plant (Ragnsells, Malmö, Sweden) (Figure 1). The number of organic amendments added to the different treatments was adjusted in order to reach the same level of nitrogen (33 kg N/ha, corresponding to a low fertilization dose for baby leaf production), with the exception of the control treatment that contained only soil. The amounts of each type of amendment added were calculated based on the information given on their respective packaging regarding nitrogen content (%), and adjustments were made for differences in mineralization rates (chicken manure: 4.1%, 9 g/pot; cow manure: 0.05%, 228 g/pot; sludge: 5.3%, 7 g/pot). The amendments were weighed separately for each replicate pot, and thoroughly mixed with the soil, up to pot capacity. Water was added to increase moisture levels in the soil, and all pots were covered with black plastic in order to avoid germination of weed seeds. The soil mixtures were incubated in a greenhouse chamber for 28 days at 22 °C, with a relative humidity of 60%. Samples were collected at the start of the experiment (i.e., time point 0, representing the amendment itself before mixing with soil) from the different types of organic amendments and the soil separately. The incubated mixtures were sampled on day 1, 7, and 28. For each sampling event, four biological replicates were used. For each replicate, the contents of an entire pot were poured into a plastic container where it could be thoroughly mixed. From this mixture three 50 mL Falcon tubes were filled and placed in a −20 °C freezer before DNA extraction.

### 4.2. DNA Extraction and Droplet Digital PCR Analysis of Microbiota (16S rRNA) and ARG

DNA was extracted from the soil samples (250 mg) as well as the organic amendments separately at four different time points using the ZymoBIOMICS DNA Miniprep Kit (Zymo Research, Irvine, CA, USA) according to the manufacturer’s instructions. Absolute quantification of gene copies was conducted on a Bio-Rad QX200 Digital Droplet system according to the manufacturer’s instructions, using general primers targeting the prokaryotic 16S rRNA gene and primers specific to *Bdellvibrio*, *Bacteriovorax*, and the ARGs *tetM*, *tetA*, and *vanA* (Table 2). The PCR was conducted in a Bio-Rad C1000 thermal cycler, following standard ddPCR cycling settings as recommended by the manufacturer. Amplified products were analyzed in a QX200 Droplet Reader (Bio-Rad, Hercules, CA, USA), and the data were analyzed with the QuantaSoft analysis software (v1.7). The results were converted into gene copies per gram of soil. All samples were analyzed in biological (n = 4) and technical (n = 3 per biological replicate) replicates. For all primer/probe pairs, negative controls were conducted (milliQ water, triplicate) that always displayed zero gene copies in the analysis. None of the primer/probe pairs generated positive signals from single-strain microbial DNA, indicating specificity to their targets. Gene copy numbers were only considered relevant when all replicates (triplicates) had a signal, in order to minimize false reporting of background noise despite taking into account the lack of signal in the negative controls. No positive samples contained less than 1 gene copy per 10 μL of extracted DNA, which was then calculated back to correspond to gram soil.

### 4.3. Nutrient Analysis of Soil

Soil samples (0.5 kg) were taken at days 0 and 28 from the control treatment and at day 28 from the treatments containing soil and organic amendments. From all treatments, pooled samples of all four replicates were sent for Spurway analysis to a certified commercial laboratory (LMI AB, Helsingborg, Sweden) in order to determine plant-available nutrients (nitrate, ammonium, phosphorus, potassium, magnesium, sulfur, calcium, manganese, boron, iron, sodium, and aluminum), pH, and electrical conductivity (EC).

### 4.4. Statistical Analysis

Statistical analyses were performed using GraphPad Prism 9.4.0. Unpaired t-test was used for group comparisons between various manures, whereas paired t-test was used for group comparisons within the same manure but between different time-points. For correlation analyses, Spearman’s correlation was used. Mean values from three technical replicates were used as input for statistical analyses. *p*-values were considered significant at *p* < 0.05, with * *p* < 0.05, ** *p* < 0.01, and *** *p* < 0.001.

## Figures and Tables

**Table 1:** Spurway analysis of plant available nutrients in the control treatment (only soil) at day 0 and 28, as well as the soil mixed with organic amendments at day 28.

Analysis	Unit	Soil (d0)	Soil (d28)	Chicken	Cow	Sludge
pH		5.8	6.0	5.85	6.35	5.45
Electrical conductivity	mS/cm	3.4	2.75	3.8	2.45	2.9
Nitrogen (N)	mg/L	160	145	180	140	210
Nitrate (N)	mg/L	160	145	180	140	210
Ammonium (N)	mg/L	3	1	1	1	1
Phosphorous (P)	mg/L	13	15	23	110	28.5
Potassium (K)	mg/L	52	54	130	420	88
Magnesium (Mg)	mg/L	78	77	91.5	135	50
Sulfur (S)	mg/L	250	145	230	73.5	135
Calcium (Ca)	mg/L	790	675	715	510	705
Manganese (Mn)	mg/L	1.9	0.66	1.5	0.95	1.65
Boron (B)	mg/L	0.54	0.6	0.6	1.2	1.05
Iron (Fe)	mg/L	1.3	1.8	1.35	1.55	1.58
Sodium (Na)	mg/L	180	170	205	105	72.5
Aluminum (Al)	mg/L	3.2	4.25	3.15	3.1	4.5

**Table 2:** ddPCR primers used within the study.

Target	Forward Primer	Reverse Primer	Probe	Ref
*16S*	AGAGTTTGATCCTGGCTCAGGA	CGTGTTACTCACCCGTCCG	CGCTGGCGGCGTGCCTAATACATGC	[34,35]
*Bdellovibrio*	GGAGGCAGCAGTAGGGAATA	GCTAGGATCCCTCGTCTTACC	CGCGTGAGTGATGAAGGCCTTCGGGTCG	This study
*Bacteriovorax*	CAGCCGCGGTAATACGAA	CGGATTTTACCCCTACATGC	GGGTGCAAGCGTTGTTCGGATTTATTGGGC	This study
*tetA*	TTGAACGGCCTCAATTTCCT	GATGAAGAAGACCGCCATCA	GCATGACCGTCGTCGCCGCCC	[34,35,36]
*tetM*	TGCAAGAAAAGTATCATGTGGAG	AAACCGAGCTCTCATACTGC	TGCCGCCAAATCCTTTCTGGGCTTCCA	[34,35,36]
*vanA*	GTTGTGCGGTATTGGGAAAC	GTTTCCTGTATCCGTCCTCG	GCCGCGTTAGCTGTTGGCGAGGT	This study

## Data Availability

All data are available within the manuscript.

## Supplementary Materials

The following supporting information can be downloaded at: https://www.mdpi.com/article/10.3390/antibiotics13080750/s1, Table S1, Correlation matrix.
